# Assisted reproductive technology represents a possible risk factor for development of epimutation-mediated imprinting disorders for mothers aged ≥ 30 years

**DOI:** 10.1186/s13148-020-00900-x

**Published:** 2020-07-22

**Authors:** Kaori Hara-Isono, Keiko Matsubara, Masashi Mikami, Takahiro Arima, Tsutomu Ogata, Maki Fukami, Masayo Kagami

**Affiliations:** 1 Department of Molecular Endocrinology, National Research Institute for Child Health and Development, 2-10-1 Okura, Setagaya-ku, Tokyo, 157-8535 Japan; 2 Department of Pediatrics, Keio University School of Medicine, 35 Shinanomachi, Shinjuku-ku, Tokyo, 160-8582 Japan; 3 Division of Biostatistics, Clinical Research Center, National Center for Child Health and Development, 2-10-1 Okura, Setagaya-ku, Tokyo, 157-8535 Japan; 4 Department of Informative Genetics, Environment and Genome Research Center, Tohoku University Graduate School of Medicine, 2-1 Seiryo-cho, Aoba-ku, Sendai, 980-8575 Japan; 5 Department of Pediatrics, Hamamatsu University School of Medicine, 1-20-1 Handayama, Higashi-ku, Hamamatsu, 431-3192 Japan

**Keywords:** Assisted reproductive technology, Imprinting disorders, Epimutation, Maternal age, Risk factors

## Abstract

**Backgrounds:**

The proportion of assisted reproductive technology (ART)-conceived livebirths of patients with imprinting disorders (IDs) is higher than that of the general population. Whether this is due to ART or confounding effects of advanced parental age was not investigated. We examined the association of ART and parental ages at childbirth for the development of eight epimutation-mediated imprinting disorders (epi-IDs).

**Results:**

We enrolled 136 patients with epi-IDs and obtained general population ART data from the Japanese robust nationwide registry. We compared the proportion of ART-conceived livebirths and maternal childbearing ages between patients with epi-IDs and the general population. The proportion of ART-conceived livebirths in patients with epi-IDs was higher than that in mothers aged ≥ 30 years, the age group in which more than 90% of ART procedures performed. The maternal childbearing ages of patients with epi-IDs were widely distributed from 19 to 45 (median: 32) within the approximate 2.5th to 97.5th percentiles of maternal childbearing ages of the general population. In addition, we compared the proportion of ART-conceived livebirths and parental ages at childbirth across patients with eight epi-IDs. We demonstrated that more than 90% of ART-conceived patients with epi-IDs were found in Silver-Russell syndrome (SRS) and Beckwith-Wiedemann syndrome (BWS) patients, and parental ages were almost consistent in patients with eight epi-IDs, except Prader-Willi syndrome.

**Conclusions:**

According to the prerequisite that most of the ART procedures in Japan are performed on mothers aged ≥ 30 years, ART can be a risk factor for the development of epi-IDs, particularly SRS and BWS, for mothers aged ≥ 30 years.

## Background

Assisted reproductive technology (ART) is widespread in developed countries [1]. In Japan, 54,110 livebirths, accounting for one in 18.1 neonates, were born using ART in 2016 [2]. Artificial manipulations are possible to alter epigenetic modification of gametes and embryos. In fact, several studies revealed the impact of ART procedures on methylation imprints [3, 4]. Genomic imprinting is a marking mechanism to identify parental origin for the mono-allelic expression of imprinted genes. Differentially methylated regions (DMRs) in the imprinted regions function as the imprinting control center [5]. DNA methylation at the DMRs is a critical epigenetic modification for the regulation of expression of the imprinted genes [5]. DNA methylation imprints are erased in primordial germ cells and re-established in a sex-specific manner during gametogenesis [6]. After fertilization, although global demethylation occurs, DNA methylation imprints in embryos are maintained. ART procedures may affect this genomic imprinting in gametogenesis and embryonic development in the preimplantation stage in several ways. Controlled ovarian stimulation (COS) and in vitro maturation (IVM) of oocytes can interfere with acquisition of maternal genomic imprinting during oogenesis. In fact, superovulated human oocytes showed aberrant methylation levels of the *H19*-DMR and *MEST*-DMR [7], and IVM-derived oocytes also showed aberrant methylation levels of the *KCNQ1OT1*-DMR [8]. In vitro fertilization (IVF), intracytoplasmic sperm injection (ICSI), and embryo culture can interfere with the maintenance of genomic imprinting in the early embryos. More than 50% of cultured preimplantation day three embryos and blastocysts showed aberrant methylation levels of the *H19-*, *SNRPN*-, and *KCNQ1OT1*-DMRs, demonstrating that ART-derived embryos possessed a high frequency of imprinted methylation errors [9]. In addition, cord blood and placentas from pregnancies conceived by IVF and ICSI had higher methylation levels of the *PLAGL1*-DMR [10] and lower methylation levels of the *H19*-DMR and *MEST*-DMR [11], respectively, compared with those from pregnancies conceived by natural conception. These studies suggested that IVF and ICSI altered the epigenetic signatures of offspring. However, it remains unclear whether ART procedures directly affect methylation imprints, or whether the parental issues concerned with ART, such as infertility and advanced parental age, lead to abnormal methylation. Indeed, impairment of sperm DNA methylation in male infertility and the correlation between parental advanced age at childbirth and levels of DNA methylation in the offspring have been reported [12, 13].

Imprinting disorders (IDs) are clinical syndromes associated with disruption of imprinted gene expression [6]. The etiologies of IDs are pathogenic variants in causative genes, copy number variants (CNVs) involving the imprinted regions, uniparental disomy (UPD) of chromosomes having imprinted genes, and epigenetic changes of the disease-responsible DMRs, i.e., epimutation. The aberrant methylation at the paternally methylated germline-derived DMRs causes Silver-Russell syndrome (SRS), Beckwith-Wiedemann syndrome (BWS), Kagami-Ogata syndrome (KOS), and Temple syndrome (TS14). On the other hand, aberrant methylation at the maternally methylated germline-derived DMRs causes BWS, Prader-Willi syndrome (PWS), Angelman syndrome (AS), pseudohypoparathyroidism 1B (PHP1B), and transient neonatal diabetes mellitus (TNDM). Previous epidemiological studies showed that the proportion of pregnancies conceived by ART was higher in patients with SRS, BWS, PWS, and AS than in the general population [14, 15]. Previous reports have some limitations, as follows: (1) there was no investigation for the associations between ART and the remaining four IDs such as KOS, TS14, PHP1B, and TNDM; (2) there was no study which focused only on epimutation-mediated imprinting disorders (epi-IDs); (3) the confounding effect of advanced parental age at childbirth remains to be elucidated. To clarify whether ART or the confounding effect of parental age at child birth facilitates the development of epi-IDs, we examined (1) the proportion of ART-conceived livebirths and the distribution of maternal childbearing ages in patients with epi-IDs and the general population for each year and (2) the proportion of ART-conceived livebirths and parental ages at childbirth across patients with eight epi-IDs.

## Results

### Numbers of the patients with epi-IDs

We enrolled 136 patients with epi-IDs confirmed by molecular studies as described in methods and obtained their clinical information about parental age, conception (naturally or ART-conceived), and ART methods utilized in ART-conceived patients. According to the definition used in the Japan Society of Obstetrics and Gynecology (JSOG) database, we classified patients conceived with IVF, ICSI, and frozen embryo transfer (FET) as ART-conceived livebirths and patients born after COS only were not included in ART-conceived livebirths. The numbers of the patients with eight epi-IDs and affected DMRs are shown in Table 1. Of note, we included 31 BWS patients consisting of nine patients with the hypermethylated *H19/IGF2*:IG-DMR (BWS subgroup-1) and 22 patients with the hypomethylated *KCNQ1OT1*:TSS-DMR (BWS subgroup-2). Of 136 patients with epi-IDs, information about paternal age and maternal age at childbirth were obtained from 131 and 134 patients, respectively.

**Table 1:** The numbers of the patients with eight representative epi-IDs and affected DMRs

epi-IDs	Affected DMRs	*n*
Silver-Russell syndrome	*H19/IGF2*:IG-DMR hypomethylation	77
Beckwith-Wiedemann syndrome	*H19/IGF2*:IG-DMR hypermethylation	9
	*KCNQ1OT1*:TSS-DMR hypomethylation	22
Kagami-Ogata syndrome	*MEG3/DLK1*:IG-DMR and *MEG3*:TSS-DMR hypermethylation	5
Temple syndrome	*MEG3/DLK1*:IG-DMR and *MEG3*:TSS-DMR hypomethylation	4
Prader-Willi syndrome	*SNURF*:TSS-DMR hypermethylation	4
Angelman syndrome	*SNURF*:TSS-DMR hypomethylation	5
Pseudohypoparathyroidism 1B	*GNAS A/B*:TSS-DMR hypomethylation	8
Transient neonatal diabetes mellitus	*PLAGL1*:alt-TSS-DMR hypomethylation	2
Total		136

*epi-IDs* epimutation-mediated imprinting disorders, *DMRs* differentially methylated regions

### Characteristics of birth data of the Japanese general population

**Fig. 1:**
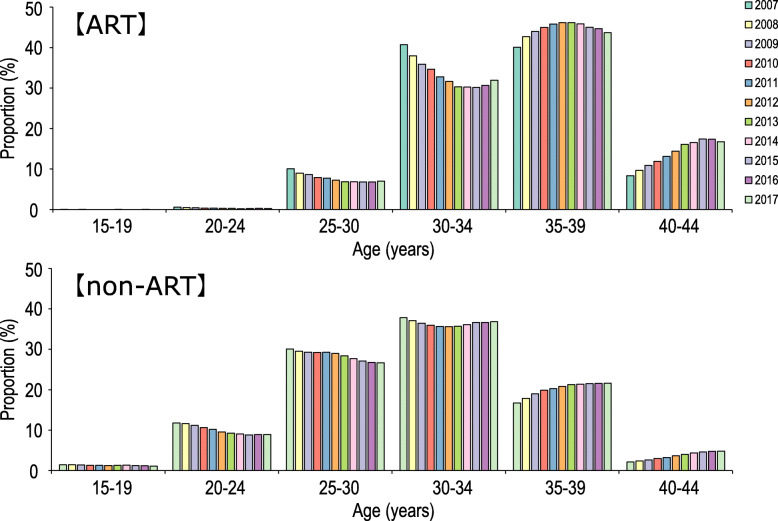
The proportion of maternal childbearing ages of ART pregnancy and that of non-ART pregnancy in Japan between 2007 and 2017. ART, assisted reproductive technology

We combined the data obtained from the Annual Nationwide Survey Data from the Ministry of Health, Labor (MHLW) and those from the JSOG and revealed the trends of ART pregnancy and maternal childbearing age of the Japanese general population. The approximate median and 2.5th to 97.5th percentiles of maternal childbearing age were calculated using MHLW database as described in methods. Figure 1 shows the distribution of maternal childbearing ages of ART pregnancy and that of non-ART pregnancy in Japan between 2007 and 2017. Maternal childbearing ages in both groups had become higher year by year. Maternal childbearing age of the most common group in ART pregnancy (35–39 years old) was higher than that in non-ART pregnancy (30–34 years old). In ART pregnancy, the proportion of mothers aged ≥ 30 years was more than 90% and that of mothers aged < 30 years was only 7%. On the other hand, the proportion of mothers aged ≥ 30 years was about 60% in non-ART pregnancy.

### Comparison of the proportion of ART-conceived livebirths and maternal childbearing ages between patients with epi-IDs and the general population

**Fig. 2:**
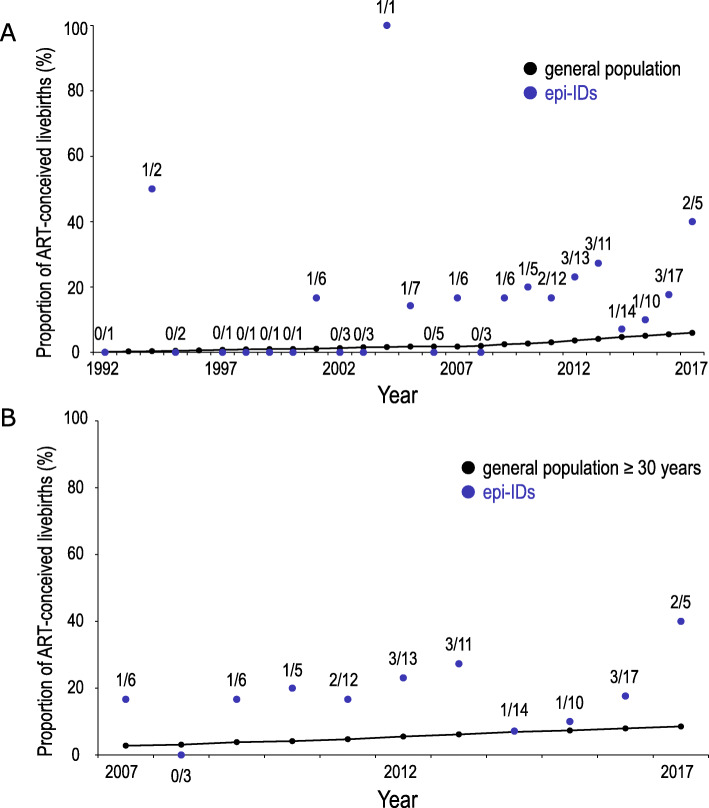
The proportion of ART-conceived livebirths in the general population and that in patients with epi-IDs. Blue dots indicate the proportion of ART-conceived livebirths in patients with epi-IDs with actual numbers. **a** Between 1992 and 2017. Black dots represent the proportion of ART-conceived livebirths in the general population. **b** Between 2007 and 2017. Black dots indicate the proportion of ART-conceived livebirths in the general population of maternal age ≥ 30 years. ART, assisted reproductive technology; epi-IDs, epimutation-mediated imprinting disorders

**Fig. 3:**
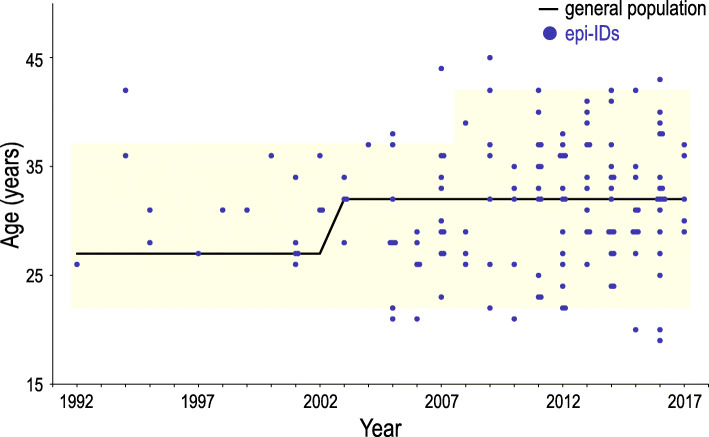
The distribution of maternal childbearing ages in the patients with epi-IDs between 1992 and 2017. Blue dots indicate the maternal ages of the patients with epi-IDs in each year. The black line indicates the median age of the general population and the yellow area indicates the 2.5th to 97.5th percentiles of the general population. epi-IDs, epimutation-mediated imprinting disorders

To clarify whether ART and advanced childbearing age bear the risk for the development of epi-IDs, we compared (1) the proportion of ART-conceived livebirths and (2) the distribution of the maternal childbearing ages, between patients with epi-IDs and the general population. Of 136 patients with epi-IDs, 22 patients (16.2%) were conceived with ART. In ART-conceived patients, 12, six, and four patients were born from IVF, ICSI, and FET, respectively. Figure 2a shows the comparison of the proportion of ART-conceived livebirths in the general population and that in patients with epi-IDs every year from 1992 to 2017. The proportion of ART-conceived livebirths was higher in the patients with epi-IDs than that in the general population, particularly from 2004 to 2017 (Fig. 2a). Because the proportion of mothers aged ≥ 30 years was more than 90% in ART pregnancy, we compared the proportion of ART-conceived livebirths in all patients with epi-IDs and that in the general population of childbearing age ≥ 30 years from 2007 to 2017 when we could obtain the age distribution of mothers who conceived with ART from the JSOG database. Patients with epi-IDs showed a high proportion of ART-conceived livebirths when compared to the general population of maternal age ≥ 30 years (Fig. 2b). Figure 3 shows the distribution of maternal childbearing ages in the patients with epi-IDs between 1992 and 2017. The approximate median maternal childbearing age of the general population was 27 years old until 2002, then up to 32 years old in 2003 and beyond. The maternal childbearing ages of the patients with epi-IDs varied widely from 19 to 45 with the median age of 32. In addition, most of the maternal childbearing ages of the patients with epi-IDs were distributed within the approximate 2.5th to 97.5th percentiles of maternal childbearing ages of the general population.

### Comparison of the proportion of ART-conceived livebirths and parental ages at childbirth across patients with eight epi-IDs

**Fig. 4:**
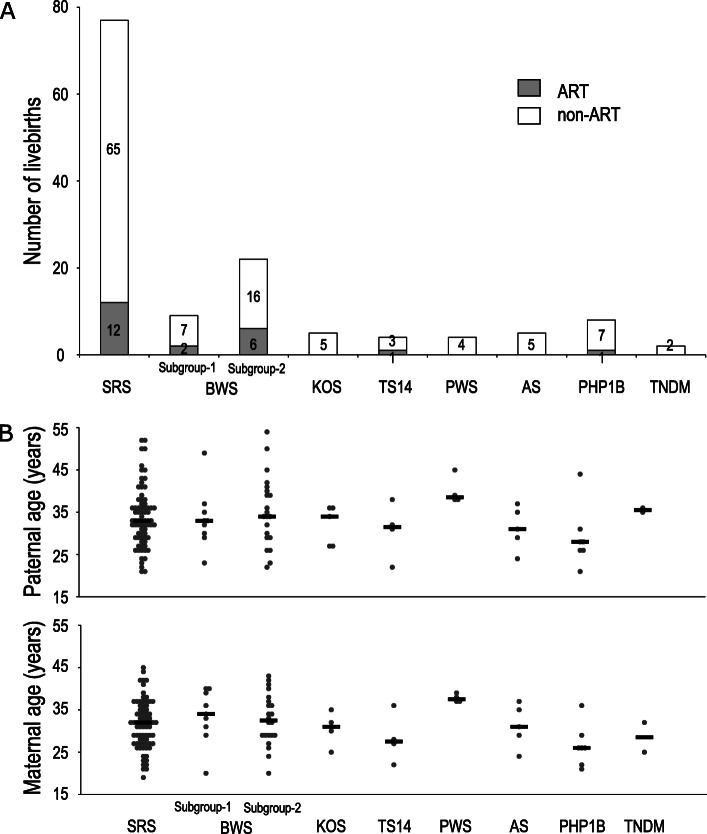
Comparison across patients with eight epi-IDs. **a** The proportion of ART-conceived livebirths in patients with eight epi-IDs. Gray and white bars indicate the number of ART-conceived and non-ART-conceived livebirths in patients with each epi-ID, respectively. **b** The distribution of paternal and maternal ages at childbirth in patients with eight epi-IDs. Black bars indicate the median paternal or maternal age of patients with each epi-ID. ART, assisted reproductive technology; epi-IDs, epimutation-mediated imprinting disorders; SRS, Silver-Russell syndrome; BWS, Beckwith-Wiedemann syndrome; KOS, Kagami-Ogata syndrome; TS14, Temple syndrome; PWS, Prader-Willi syndrome; AS, Angelman syndrome; PHP1B, pseudohypoparathyroidism 1B; TNDM, transient neonatal diabetes mellitus

To investigate the effect of ART and parental age for the development of epi-IDs in detail, we compared the proportion of ART-conceived livebirths and parental ages at childbirth across patients with eight epi-IDs. Figure 4 shows the number of ART-conceived livebirths and the distribution of parental ages at childbirth in patients with eight epi-IDs. ART-conceived livebirths were identified in patients with SRS (15.6%), BWS (25.8%; subgroup-1 [22.2%], subgroup-2 [27.3%]), TS14 (25.0%), and PHP1B (12.5%). Twenty out of 22 (90.9%) ART-conceived livebirths in epi-IDs were patients with SRS or BWS. On the contrary, ART-conceived livebirth was not found in KOS, PWS, AS, and TNDM. Both the medians of paternal and maternal age at childbirth were consistent in patients with eight epi-IDs, except PWS (Fig. 4b). Notably, in PWS patients, the distributions of paternal (range 38–45) and maternal ages (range 37–39) at childbirth were higher than those in the other epi-IDs, although only four PWS patients were included in this study.

## Discussion

This study is the first trial to evaluate the association of ART and parental age at childbirth for the development of IDs focusing on epimutation. We included 136 patients with epi-IDs, the largest study population enrolled so far, and performed cross-sectional analysis of eight representative epi-IDs, for the first time. Furthermore, we utilized the robust data from a nationwide ART registry system, which was characterized by its mandatory reporting system and high compliance rate [2].

Comparison between patients with epi-IDs and the general population revealed several notable findings. First, we found a higher prevalence of ART-conceived livebirths in patients with epi-IDs than in the general population (Fig. 2a). In Japan, more than 90% of ART-conceived livebirths were born from mothers aged ≥ 30 years (Fig. 1). Therefore, when we consider the impacts of ART for the development of epi-IDs, we can investigate its effects only for mothers aged ≥ 30 years. According to this prerequisite, we compared the proportion of ART-conceived livebirth of patients with epi-IDs to that of the general population of maternal childbearing age ≥ 30 years (Fig. 2b). Patients with epi-IDs including the mothers of all ages showed a high proportion of ART-conceived livebirths when compared to the general population of maternal childbearing age ≥ 30 years. Thus, we demonstrate that ART performed on mothers aged ≥ 30 is likely to facilitate the epi-IDs. Second, the maternal childbearing ages of patients with epi-IDs were widely distributed within the 2.5th to 97.5th percentiles of that of the general population (Fig. 3). Conversely, a previous study about UPD-mediated PWS (maternal UPD of chromosome 15) revealed that the distribution of maternal childbearing ages was significantly skewed to the advanced ages and implied that the advanced childbearing age was a predisposing factor for the development of UPD because of increased meiotic errors [16]. Unlike UPD-mediated IDs, there seemed to be no association between the development of epi-IDs and maternal childbearing age. This fact is compatible with a recent study in which advanced maternal age had no effect on imprinted methylation acquisition in mouse oocytes and postzygotic imprinted methylation maintenance in mouse embryos [17].

Several matters should be pointed out regarding comparison of the proportion of ART-conceived livebirths and parental ages across patients with eight representative epi-IDs. First, we compared our results to the previous studies investigating the proportions of ART-conceived livebirths in IDs (Table 2) [14, 18–21]. Only our study included all eight representative IDs focusing on epimutations and performed molecular analyses in all patients. The proportions of ART-conceived livebirths in epimutation-mediated SRS (15.6%) and BWS (25.8%) in our study were higher than the previously reported proportion of ART-conceived livebirths in SRS and BWS including all genetic causes (Table 2). We speculate that the methylation status of SRS and BWS related DMRs (*H19/IGF2*:IG-DMR, *KCNQ1OT1*:TSS-DMR) is particularly vulnerable to the effects of ART. This finding raises the possibility that epimutations of SRS and BWS related DMRs are more susceptible to the effect of ART than other genetic etiologies, such as UPD of chromosomes 7 and 11. In our study, approximately 90% of ART-conceived livebirths in epi-IDs were found in SRS and BWS, whereas previous studies showed that about 30 to 50% of ART-conceived livebirths in IDs were found in SRS and/or BWS (Table 2). This result is due to the fact that epimutation is the most common genetic cause of SRS and BWS (Table S1) and previous studies included patients with genetic causes other than epimutations resulting in IDs. Indeed, patients with SRS or BWS accounted for approximately 80% of the patients included in our study. Unlike previous studies, we found no ART-conceived livebirths in epimutation-mediated PWS and AS patients (Table 2). This could be explained by the rarity of epimutation in PWS (< 1%) and AS (< 3%) patients. Second, we evaluated the proportion of ART-conceived livebirths in KOS, TS14, PHP1B, and TNDM and found two ART-conceived livebirths, one in TS14 and one in PHP1B. We previously reported two ART-conceived livebirths with TS14, one in epimutation and one in UPD [22]. Regarding PHP1B, only two livebirths following ART had been reported and all of them were caused by the loss of methylation at *GNAS A/B* locus (epimutation) [23, 24]. These results might reflect the association between ART and epimutation, particularly in TS14 and PHP1B. Third, parental age at childbirth was consistent in eight epi-IDs except PWS. This may be explained by the assumption that parental age at childbirth has a small or no effect on the specific DMRs. In fact, a previous study in fetal cord blood of healthy infants revealed that parental age at childbirth has almost no effect on the methylation levels of the DMRs [25]. Additionally, both paternal and maternal age at childbirth in PWS patients were higher than those in the other epi-IDs. Because we included a limited number of epimutation-mediated PWS patients, further accumulation of these patients could clarify the association between advanced parental age at childbirth and epimutation in PWS patients.

**Table 2:** Summary of the studies investigating the proportions of ART-conceived livebirths in imprinting disorders

IDs	Country	Number of patients	Number of patients with molecular analysis	Non-ART	ART (%)	Genetic causes found in ART-conceived IDs	Interventions of ART performed
Sutcliffe, 2006* [18]	UK						
Total		317	12^a^	295	22		IVF, ICSI
BWS		79	8	68	11 (13.9)	epi, 8^d^; not doing genetic testing, 3	
PWS		163	2	154	9 (5.5)	del, 2; not doing genetic testing, 7	
AS		75	2	73	2 (2.7)	epi, 1; del, 1	
Doornbos 2007* [19]	Netherlands						
Total		220	11^a^	206	14		IVF, ICSI,
BWS		71	6	65	6 (8.5)	epi, 6^d^	COS, IUI
PWS		86	3	82	4 (4.7)	del, 3; not doing genetic testing, 1	
AS		63	2	59	4 (6.3)	del, 2; not doing genetic testing, 2	
Tenorio 2016* [20]	Spain						
BWS		156	156^b^	139	17 (10.9)	epi, 15^d^; unknown, 2	IVF, AI, COS
Mussa, 2017* [21]	Italy						
BWS		38	7^a^	31	7 (18.4)	epi, 3^d^; UPD, 2; unknown, 2	IVF, ICSI, IUI
Hattori, 2019* [14]	Japan						
Total		931	556	888	43		IVF, ICSI
SRS		67	22^c^	59	8 (11.9)	epi, 5; not doing genetic testing, 3	
BWS		117	43	110	7 (6.0)	epi, 4^d^; not doing genetic testing, 3	
PWS		520	366	496	24 (4.6)	epi, 6; del, 6; UPD, 9; not doing genetic testing, 3	
AS		227	147	223	4 (1.8)	del, 4	
This study**	Japan						
Total		136	136^b^	114	22		IVF, ICSI, FET
SRS		77	77	65	12 (15.6)	epi, 12	
BWS		31	31	23	8 (25.8)	epi, 8^e^	
KOS		5	5	5	0		
TS14		4	4	3	1 (25.0)	epi, 1	
PWS		4	4	4	0		
AS		5	5	5	0		
PHP1B		8	8	7	1 (12.5)	epi, 1	
TNDM		2	2	2	0		

*IDs* imprinting disorders, *ART* assisted reproductive technology, *BWS* Beckwith-Wiedemann syndrome, *PWS* Prader-Willi syndrome, *AS* Angelman syndrome, *SRS* Silver-Russell syndrome, *KOS* Kagami-Ogata syndrome, *TS14* Temple syndrome, *PHP1B* pseudohypoparathyroidism 1B, *TNDM* transient neonatal diabetes mellitus, *UK* United Kingdom, *epi* epimutation, *del* deletion, *UPD* uniparental disomy, *IVF* in vitro fertilization, *ICSI* intracytoplasmic sperm injection, *COS* controlled ovarian stimulation, *IUI* intrauterine insemination, *AI* artificial insemination, *FET* frozen embryo transfer

*Patients with all genetic causes resulting in IDs were included

**Patients with only epimutation-mediated IDs were included

^a^Molecular analysis was performed in patients with ART-conceived IDs only

^b^Molecular analysis was performed in all patients

^c^Including 10 patients from our study

^d^All patients had hypomethylated *KCNQ1OT1*:TSS-DMR

^e^Patients had either hypomethylated *KCNQ1OT1*:TSS-DMR or hypermethylated *H19/IGF2*:IG-DMR

Our study has some limitations. First, the number of patients with epi-IDs was obviously much lower (20,000-fold) than that in the general population. Particularly, we included the limited number of patients with epi-IDs other than SRS and BWS. Because our subjects were patients referred to us for genetic testing for IDs, we did not include all patients with epi-IDs in Japan. Thus, we could not perform statistical tests for the comparison of the proportions of ART-conceived livebirths between patients with epi-IDs and the general population. These are inevitable limitations of our study, which focuses on rare diseases. Second, as in previous reports, we could not perform statistical analysis to determine whether epimutation is caused by ART or the confounding effect of advanced maternal childbearing age. Because mothers who received ART were mostly of advanced age in Japan, it is impossible to estimate the independent effects of ART itself. In addition, we could not evaluate whether the interaction between ART and maternal childbearing age induces an increase in epi-IDs. Third, the confounding effect of infertility was not evaluated, as we did not inquire about the reason for using ART in the questionnaire. Lastly, the effect of COS alone was not investigated, as we did not include COS into the ART procedure based on the JSOG’s definition. A previous study revealed that COS interfered with maternal genomic imprinting in oocytes [7]. Further research is required to elucidate the effects of COS for the development of epi-IDs.

## Conclusions

In summary, based on the prerequisite that most of the ART procedures in Japan are performed on mothers aged ≥ 30 years, we concluded that ART can be a risk factor for the development of epi-IDs, especially SRS and BWS, for mothers aged ≥ 30 years.

## Methods

### Patients

We enrolled 136 patients with epi-IDs confirmed by molecular studies including 21 previously reported patients (16 SRS patients, one KOS patient, and four TS14 patients) [22, 26–28]. Patients with seven IDs other than SRS were subjected to genetic testing. Regarding SRS, we included the patients with hypomethylation of the *H19/IGF2*:IG-DMR who were suspected to have SRS due to SRS phenotype and/or severe growth failure. All patients were born from 1992 to 2017 and recruited from 2004 to 2019. Clinical information about parental age, conception (naturally or ART-conceived), and ART methods utilized in ART-conceived patients were obtained from the attending physicians by questionnaire. Because the JSOG has the database of the number of ART-conceived livebirths from IVF, ICSI, and FET, we classified patients conceived with IVF, ICSI, and FET as ART-conceived livebirths. Therefore, patients born after COS only were not included in ART-conceived livebirths.

### Birth data of the Japanese general population

The total number of livebirths and the distribution of maternal childbearing ages were obtained from the Annual Nationwide Survey Data from the MHLW (http://www.mhlw.go.jp/toukei/list/81-1.html). This database showed the number of livebirths by five-year maternal age groups (≤ 14 years, 15–19 years, 20–24 years, 25–29 years, 30–34 years, 35–39 years, 40–44 years, 45–49 years, and ≥ 50 years). Thus, we could not obtain the exact median age and 2.5th to 97.5th percentiles of the maternal childbearing ages of the general population from this database. In this regard, because median maternal childbearing age was included in the group of 25–29 years (1992–2002) and 30–34 years (2003–2017), we considered the approximate median maternal childbearing age as 27 years (1992–2002) and 32 years (2003–2017), respectively. Likewise, we considered the approximate 2.5th percentile for childbearing age as 22 years and 97.5th percentile for childbearing age as 37 years (1992–2007) and 42 years (2008–2017).

ART data, including the number of ART-conceived livebirths after ICSI (from 1985), IVF (from 1985), and FET (from 1989), and the age distribution of mothers who conceived with ART (from 2007) were obtained from an online registration system of the JSOG (https://plaza.umin.ac.jp/~jsog-art/). This database includes data from almost all ART facilities and implemented ART cycles nationwide (604 facilities and 447,790 cycles in 2016) [2]. Detailed information collected from the registry has been reported previously [29].

### Comparison of the proportion of ART-conceived livebirths and parental ages

We compared (1) the proportion of ART-conceived livebirths and (2) maternal childbearing ages in each year between patients with epi-IDs and the general population. The birth data of the general population was obtained from registry data of MHLW and JSOG as the control. Furthermore, we also compared the proportion of ART-conceived livebirths and parental ages among patients with eight epi-IDs.

### Molecular studies

To detect patients with epimutation, we combined four genetic analysis as follows: (1) methylation analysis using pyrosequencing [28]; (2) methylation-specific multiplex ligation-dependent probe amplification (MS-MLPA, MRC Holland, Amsterdam, Netherlands) analysis utilizing commercially available MLPA probe mix for multiple segments on chromosomes 11 (ME030), 15 (ME028), and 20 (ME031); (3) microsatellite analysis for chromosomes 6 [30], 14 [31], 15 [16], and 20 [32] using patients’ and their parental genomic DNA samples; and (4) an array-based comparative genomic hybridization (aCGH) analysis using a custom-built array involving the imprinted regions related to IDs (Design ID 032112, Agilent Technologies, Santa Clara, CA, USA) [33]. The diagnostic process of each epi-ID is shown in Supplementary Figure S1.

### Statistical analysis

For the comparison of the distribution of maternal childbearing ages between patients with epi-IDs and the general population, we used the median and 2.5th and 97.5th percentiles for continuous variables as summary statistics.

## Supplementary information

**Additional file 1: Table S1.** Frequency of epimutation in the eight representative imprinting disorders.

**Additional file 2: Figure S1.** The diagnostic process of eight epimutation-mediated imprinting disorders.

## Data Availability

All data generated or analyzed during this study are available from the corresponding author on reasonable request.

## Abbreviations

aCGH: Array-based comparative genomic hybridization
ART: Assisted reproductive technology
AS: Angelman syndrome
BWS: Beckwith-Wiedemann syndrome
CNVs: Copy number variants
COS: Controlled ovarian stimulation
DMRs: Differentially methylated regions
epi-IDs: Epimutation-mediated imprinting disorders
FET: Frozen embryo transfer
ICSI: Intracytoplasmic sperm injection
IDs: Imprinting disorders
IVF: In vitro fertilization
IVM: In vitro maturation
JSOG: Japan Society of Obstetrics and Gynecology
KOS: Kagami-Ogata syndrome
MHLW: Ministry of Health, Labor
MS-MLPA: Methylation-specific multiplex ligation-dependent probe amplification
PHP1B: Pseudohypoparathyroidism 1B
PWS: Prader-Willi syndrome
SRS: Silver-Russell syndrome
TNDM: Transient neonatal diabetes mellitus
TS14: Temple syndrome
UPD: Uniparental disomy
