# Supplementary material for: Assisted reproductive technology represents a possible risk factor for development of epimutation-mediated imprinting disorders for mothers aged ≥ 30 years
Source: Clin Epigenetics. 2020 Jul 22;12:111. doi: 10.1186/s13148-020-00900-x (PMC7374921; doi:10.1186/s13148-020-00900-x)
Supplement: Supplementary file 2 — Additional file 2: Figure S1. The diagnostic process of eight epimutation-mediated imprinting disorders. [file 13148_2020_900_MOESM2_ESM.pdf]

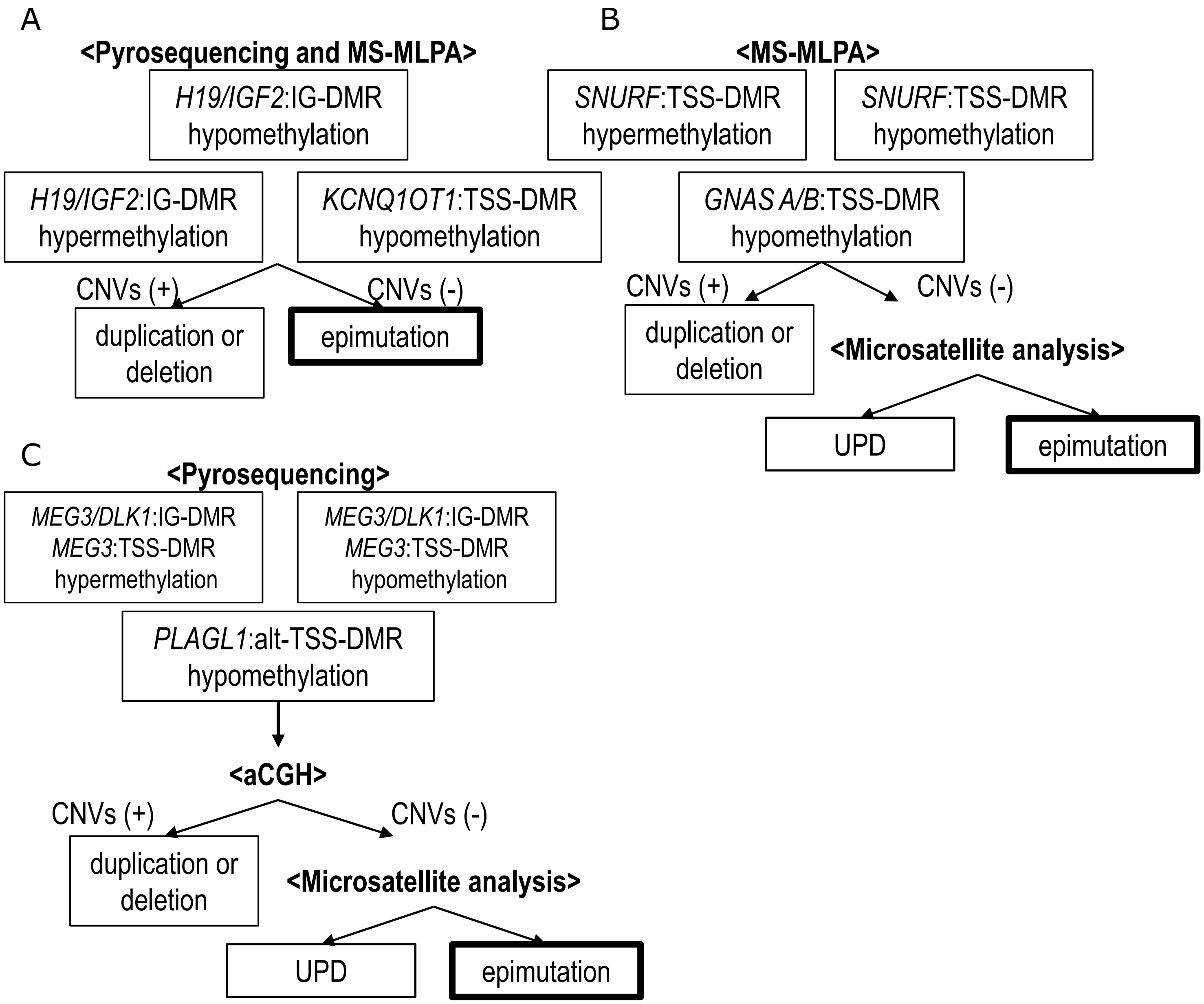

Supplementary Figure S1: The diagnostic process of eight epimutation-mediated imprinting disorders.

A. The diagnostic process of SRS and BWS patients. We performed methylation analysis using pyrosequencing for the *H19/IGF2*:IG-DMR and *KCNQ1OT1*:TSS-DMR, and MS-MLPA for the 11p15 imprinted region. When patients showed hypomethylation of the *H19/IGF2*:IG-DMR (SRS), hypermethylation of the *H19/IGF2*:IG-DMR (BWS), or hypomethylation of the *KCNQ1OT1*:TSS-DMR (BWS) without CNVs, we considered the epimutation as a genetic cause of each imprinting disorder. Because of the possibilities of having CNVs involving the 11p15 imprinted region or mosaic UPD of chromosome 11, we did not include the patients with the abnormal methylation levels of both the *H19/IGF2*:IG-DMR and *KCNQ1OT1*:TSS-DMR.

B. The diagnostic process of PWS, AS, and PHP1B patients. We first performed MS-MLPA on chromosomes 15 (for PWS and AS), and 20 (for PHP1B) to detect both methylation defects and CNVs at the disease-responsible regions. When patients had the abnormal methylation levels at disease-responsible DMRs without CNVs, we performed microsatellite analysis to exclude UPD, then confirmed the epimutation as a genetic cause.

C. The diagnostic process of KOS, TS14, and TNDM patients. We first performed pyrosequencing of the *MEG3/DLK1*:IG-DMR, *MEG3*:TSS-DMR (for KOS and TS14) and the *PLAGL1*:alt-TSS-DMR (for TNDM) to detect methylation defects of disease-responsible DMRs. When the abnormal methylation levels of the DMRs were found in patients, we performed aCGH analysis using the custom-built array to exclude CNVs, microsatellite analysis to exclude UPD, then confirmed the epimutation as a genetic cause.

MS-MLPA, methylation-specific multiplex ligation-dependent probe amplification; aCGH, array-based comparative genomic hybridization; DMR, differentially methylated region; CNVs, copy number variations; UPD, uniparental disomy; SRS, Silver-Russell syndrome; BWS, Beckwith-Wiedemann syndrome; PWS, Prader-Willi syndrome; AS, Angelman syndrome; PHP1B, pseudohypoparathyroidism 1B; KOS, Kagami-Ogata syndrome; TS14, Temple syndrome; TNDM, transient neonatal diabetes mellitus.
